# Human-Derived collagen hydrogel as an antibiotic vehicle for topical treatment of bacterial biofilms

**DOI:** 10.1371/journal.pone.0303039

**Published:** 2024-05-03

**Authors:** Evan Jarman, Jordan Burgess, Ayushi Sharma, Kate Hayashigatani, Amar Singh, Paige Fox

**Affiliations:** 1 Division of Plastic & Reconstructive Surgery, Department of Surgery, Stanford University School of Medicine, Stanford, California, United States of America; 2 Division of Plastic & Reconstructive Surgery, Veterans Affairs Palo Alto Health Care System, Palo Alto, California, United States of America; Brigham and Women’s Hospital, Harvard Medical School, UNITED STATES

## Abstract

The complexity of chronic wounds creates difficulty in effective treatments, leading to prolonged care and significant morbidity. Additionally, these wounds are incredibly prone to bacterial biofilm development, further complicating treatment. The current standard treatment of colonized superficial wounds, debridement with intermittent systemic antibiotics, can lead to systemic side-effects and often fails to directly target the bacterial biofilm. Furthermore, standard of care dressings do not directly provide adequate antimicrobial properties. This study aims to assess the capacity of human-derived collagen hydrogel to provide sustained antibiotic release to disrupt bacterial biofilms and decrease bacterial load while maintaining host cell viability and scaffold integrity. Human collagen harvested from flexor tendons underwent processing to yield a gellable liquid, and subsequently was combined with varying concentrations of gentamicin (50–500 mg/L) or clindamycin (10–100 mg/L). The elution kinetics of antibiotics from the hydrogel were analyzed using liquid chromatography-mass spectrometry. The gel was used to topically treat Methicillin-resistant *Staphylococcus aureus* (MRSA) and *Clostridium perfringens* in established Kirby-Bauer and Crystal Violet models to assess the efficacy of bacterial inhibition. 2D mammalian cell monolayers were topically treated, and cell death was quantified to assess cytotoxicity. Bacteria-enhanced *in vitro* scratch assays were treated with antibiotic-embedded hydrogel and imaged over time to assess cell death and mobility. Collagen hydrogel embedded with antibiotics (cHG+abx) demonstrated sustained antibiotic release for up to 48 hours with successful inhibition of both MRSA and *C*. *perfringens* biofilms, while remaining bioactive up to 72 hours. Administration of cHG+abx with antibiotic concentrations up to 100X minimum inhibitory concentration was found to be non-toxic and facilitated mammalian cell migration in an *in vitro* scratch model. Collagen hydrogel is a promising pharmaceutical delivery vehicle that allows for safe, precise bacterial targeting for effective bacterial inhibition in a pro-regenerative scaffold.

## Introduction

Chronic wounds are a significant concern to healthcare systems due to their clinical and financial load, with approximately 6.5 million individuals within the US affected by chronic wounds and costs exceeding $50 billion USD annually [1, 2]. The burden of chronic wounds is growing rapidly due to increasing health care costs, an aging population, and the rising incidence of comorbidities that further delay wound healing [3, 4] Statistically, only 40% of chronic wounds heal over time, presenting opportunity for bacterial colonization and infection [5]. In fact, with more than 75% of chronic wounds presenting with at least one species of foreign bacteria present [6] bacterial colonization has almost become universal in chronic wounds. Burns and acute dermal wounds left unattended also commonly present with bacterial colonization or infection. Bacterial colonization impairs wound healing by delaying processes vital for complete wound closure, such as proper immune response [7] local circulation [8] and re-epithelialization [7]. Moreover, approximately 65% of bacterial infections are associated with priorly colonized bacteria, further indicating the importance of addressing this need [9].

During wound colonization, bacteria form dense biofilms, significantly reducing host immune response efficacy and impeding physiologic healing progression [10, 11]. Bacterial biofilms are the predominant state of bacteria defined by heterogenous communities of bacteria embedded in a protective extracellular matrix composed of excreted proteins, polysaccharides, and genetic material aimed to optimize microbial defense from mechanical and chemical stimulus [12]. More generally, biofilms create a challenging environment that current standard treatments cannot effectively address. Biofilm-embedded bacteria can be more than 1000X more resistant to systemic antibiotics than planktonic bacteria [13], forcing physicians to administer broad-spectrum antibiotics systemically at significantly higher concentrations than typical therapeutic dosages. The high concentrations of systemic antibiotics may increase the risk of deleterious side effects and further exacerbate the antibiotic-resistance crisis [14]. Debridement of biofilms effectively reduces the bacterial load temporarily; however, biofilm regrowth is remarkably rapid even in combination with systemic antibiotics [15]. While several other standard therapies for chronic wounds exist, they do not successfully eradicate bacterial colonization and are only moderately effective in expediting wound healing [16, 17].

Due to their ability to address the limitations of traditional treatments, topical hydrogels are promising pharmaceutical carriers to provide local antimicrobial delivery to colonized chronic wounds. Most hydrogel polymers are highly tunable, allowing for encapsulation and customized release profiles of many pharmaceuticals [18]. Furthermore, organic polymers have the additional benefits of promoting granulation tissue formation and angiogenesis, providing relevant proteins for integration into host extracellular matrix, and generating an ideal environment for wound closure similar to traditional wound dressings [19, 20]. Overall, organic hydrogel polymers may be an ideal solution to colonized chronic wounds due to their ability to naturally expedite wound healing and inhibit biofilm formation via deliverable antimicrobials.

Previously, our lab has developed a novel human-derived, collagen-rich hydrogel (cHG) embedded with antibiotics (cHG+abx) to topically treat bacterial biofilms. This treatment has been shown to successfully disrupt *Pseudomonas aeruginosa* biofilms and decrease overall wound healing time in an *in vivo* mouse stented wound model [21]. The present study examines the use of cHG as a viable carrier for topical antibiotics to inhibit bacterial growth and induce biofilm clearance of methicillin-resistant *Staphylococcus aureus* (MRSA) and *Clostridium perfringens*. The objectives of this work were: (i) to confirm human collagen hydrogel is a viable carrier of gentamicin and clindamycin and determine the elution kinetics of these antibiotics from the gel, (ii) evaluate the antimicrobial efficacy of topical treatment with cHG+abx, and (iii) determine mammalian cell cytotoxicity with topical cHG+abx treatment. We hypothesize that topical administration of human collagen hydrogel with antibiotics will effectively eradicate multiple clinically significant bacterial biofilm phenotypes while maintaining mammalian cell viability.

## Materials and methods

### Experimental strategy

The rationale behind selecting the specific experiments in this manuscript are as follows: The Kirby-Bauer assay was chosen to estimate the time of effective antibiotic release from the hydrogel, using pre-eluted samples to mitigate agar plate dehydration effects and simulate the concentrations of antibiotic released from the hydrogel over time. Similarly, the crystal violet assay assessed hydrogel efficacy in eliminating bacterial presence after predetermined elution periods. While providing insights into the timeline of effective, bactericidal antibiotic release, this assay offered a more focused assessment of reductions in biofilm bacterial density, reflecting a model more accurate to naturally occurring biofilms. The biofilm inhibition visualization assay was implemented to quantify bacterial clearance rates from established biofilms after administering topical cHG+abx treatment and allowed direct observation of bacterial clearance. Furthermore, the mammalian cell viability assay evaluated cytotoxicity to human tissue, while the in vitro scratch assay simulated natural hydrogel usage in wound healing scenarios with bacterial presence.

### Collagen hydrogel preparation

2% collagen hydrogel solutions were prepared according to a previously established protocol [22, 23]. Human flexor digitorum profundus, flexor digitorum superficialis, and flexor pollicis longus tendons were harvested from fresh-frozen cadaveric forearms. Tendons were decellularized in 0.1% ethylenediaminetetraacetic acid (EDTA) (Thermo Fisher Scientific, Waltham, MA) for 4 h followed by 0.1% sodium dodecyl sulfate (SDS) (Thermo Fisher Scientific) in 0.1% EDTA for 24 h before washing with phosphate buffered saline (PBS) (Thermo Fisher Scientific). Tendons were then lyophilized and milled into a fine powder using a Wiley Mini Mill (Thomas Scientific, Sedesboro, NJ). Routine hematoxylin and eosin (H&E) histology and microscopy was used to ensure decellularization. 25 mg/ml collagen powder was digested in 1 mg/ml porcine-derived pepsin (Sigma Aldrich, St. Louis, MO) in HCl at 2 pH for 14 hours with agitation, then stored long term at -80° C. To prepare for use, thawed collagen solutions were lowered to 20 mg/ml collagen concentration with 10X minimum essential media (MEM) (Thermo Fisher Scientific), fetal bovine serum (FBS) (Thermo Fisher Scientific), and 10X PBS (Thermo Fisher Scientific) at a ratio of 1:2:2. Solutions were neutralized to 7.4 pH with 5N NaOH on ice and stored at 4° C for a maximum of 7 days or until use. Gelation was induced by incubation at 37° C for 40 minutes.

In making cHG+abx, antibiotics were added directly to the cHG while diluting to 20 mg/ml prior to neutralization and slowly mixed on a magnetic mixer at 4° C until homogenized. The mixture was then neutralized and stored at 4° C for a maximum of 7 days or until use. Gentamicin concentrations were prepared from 50–500 mg/L (10X-100X minimum inhibitory concentration (MIC)) and clindamycin concentrations from 10–100 mg/L (10X-100X MIC) (Thermo Fisher Scientific).

To form the hydrogel on a membrane, 10 μL of cHG+abx solution was pipetted on top of a 13 mm UV sterilized polycarbonate membrane (Millipore, Burlington, MA). Membranes were incubated at 37° C to induce gelation and transferred onto 1 mL of 1X PBS in individual wells in a 24-well plate. Pre-elution is defined by the process in which cHG+abx is allowed to elute or release the embedded antibiotics into solution prior to using the cHG+abx in an experiment. Pre-eluted membranes were used in various experiments to test the efficacy of the cHG-abx after previously releasing antibiotics for a predetermined amount of time, simulating cHG+abx antibiotic concentrations at set time points into cHG+abx treatment. To prepare these samples, hydrogel on membranes were allowed to elute into 1 mL of 1X PBS in individual wells for a predetermined amount of time.

Human samples were sourced from donor cadavers from Stanford University School of Medicine and were treated with the utmost respect in processing. An IRB was not required for this study as research involving cadaver samples does not meet the definition of human subjects research.

### Bacterial strain and growth conditions

*Clostridium perfringens* (ATCC 13124) and methicillin-resistant *S*. *aureus* (MRSA252 ATCC BAA-1720) (ATCC, Manassas, VA) were selected for this study due to their clinical significance and differing biofilm phenotypes. Both bacteria were used at mid-log growth phase in all experiments. MRSA was cultured in Tryptic Soy Broth (TSB) (Remel, San Diego,

CA) under aerobic conditions, while *C*. *perfringens* was cultured in TSB (Remel) with 5% defibrinated sheep’s blood (Thermo Fisher Scientific) under anaerobic conditions. To plate bacteria, Mueller Hinton agar (Remel) and brucella agar with 5% defibrinated sheep’s blood (Remel) were used for MRSA and *C*. *Perfringens*, respectively.

To form inoculating biofilms, an established protocol from the Center for Biofilm Engineering at Montana State University was implemented [24]. Bacterial cultures were incubated overnight in their respective conditions to 10^8^ CFU/ml. 2 μL of diluted bacterial suspension (1:100) was pipetted onto UV sterilized 13 mm polycarbonate membranes, placed on agar plates, and incubated for 24 hours to induced biofilm formation.

### Scanning electron microscopy

cHG+abx samples were imaged post gelation using scanning electron microscopy (SEM) to ensure collagen fibril formation and cross-linkage. 20 μL of 10X cHG+abx was pipetted onto a blank membrane and incubated for 40 minutes to induce gelation. Membranes were fixed for 1 hour at room temperature in 2% glutaraldehyde and 4% paraformaldehyde in sodium cacodylate. Next, samples were washed for 10 min x 2 in 0.1 M sodium cacodylate buffer and then post-fixed for 1 hour in 2% OsO4 in double-distilled water (ddH20) on a rotating shaker at 50 rpm. Samples were washed for 10 min x 2 in ddH20, and then processed through ascending ethanol washes for 10 min each at concentrations of 30%, 50%, 70%, 95%, and 100% x 2. Samples were washed for 5 min 2 times in hexamethyldisilazane and mounted on stubs, sputter coated with 5 nm of gold, and imaged at 5k, 10k, and 20k magnification at the Cell Science Imaging Facility at Stanford University.

### LCMS elution profiles

Gelled 10X MIC gentamicin and clindamycin cHG+abx membranes were placed in 1 mL of 1X PBS and allowed to elute for 0, 6, 12, 24, 48, and 72 hours; cHG-only was used as a control. The PBS from each sample was diluted 1:10 in H20 and analyzed by electrospray ionization mass spectrometry on the Waters Acquity UPLC (Millford, MA) and Thermo Exploris 240 BioPharma orbitrap mass spectrometer (Thermo Fisher Scientific). Data were acquired in FullScan MS mode with mass range 100–1000 m/z and resolution 120,000 and corrected using control samples.

### Modified Kirby-Bauer assay

Bacterial inhibition was quantified using a modified version of the established Kirby-Bauer disk diffusion assay. 200 μL of MRSA and C. *perfringens* bacterial suspension (10^8^ CFU/mL) was spread onto a 100 mm agar dish and allowed to develop for 30 min at 37° C (*C*. *perfringens* cultured in anaerobic environment). Gentamicin and clindamycin cHG+abx and cHG-only (control group) gelled on polycarbonate membranes pre-eluted in 1X PBS for predetermined time periods (0, 6, 12, 24, 36, 48, 60, 72 hours) were placed membrane-face down onto the inoculated agar, with a maximum of three membranes per 100 mm dish. MRSA and *C*. *perfringens* were treated with 100X MIC gentamicin (500 mg/L) and clindamycin (100 mg/L) respectively. Plates were incubated with treatment for 12 hours at 37° before taking bacterial inhibition diameter measurements with Fiji image analysis software. Diameter measurements were used to calculate the zone of bacterial inhibition (cm^2^). All experiments were completed in triplicate. An antibiotic-only control was not used as the antibiotic liquid absorbed into and diffused through agar plate resulting in an inaccurate zone of inhibition.

### Biofilm inhibition visualization

Biofilm membranes were produced according to the method described above. Biofilms were placed in separate wells of a 12-well plate and imaged with a brightfield microscope to quantify initial biofilm areas. Biofilm membranes were then subsequently treated with 10 μL of gentamicin and clindamycin cHG+abx and cHG-only (control group) for 12 hours at culture conditions. MRSA and *C*. *perfringens* were treated with a 10X MIC concentration of gentamicin (50 mg/L) and clindamycin (10 mg/L), respectively. At 6 hours and 12 hours of treatment, the biofilm membranes were transferred to individual wells in a 12-well plate for imaging. Bacterial biofilm membranes were stained with SYTO 61 (Invitrogen, Carlsbad, CA) according to manufacturer’s instructions and imaged with a KEYENCE BZ-X700 fluorescence microscope (KEYENCE, Osaka, Japan) to quantify bacterial viability. The bacterial density was calculated at 0, 6, and 12 hours into treatment by measuring the area with viable bacteria divided by total area of the initial biofilm (biofilm at 0 hour).

### Crystal violet assay

To further detail the efficacy timeline of cHG+abx in inhibiting bacterial biofilms, post-treatment bacterial viability was quantified with a crystal violet assay [25]. 1 mL of 10^5^ CFU/mL bacterial suspension was dispensed into individual wells of a 12-well plate and incubated for 24 hours to induce biofilm formation on the well surfaces. The supernatant was aspirated and replaced with 1 mL PBS. 100X MIC gentamicin (500 mg/L) and clindamycin (100 mg/L) cHG+abx and cHG-only (control group) membranes pre-eluted in 1X PBS for 0, 24, and 48 hours were placed in each well and treated for 12 hours in appropriate culture conditions. Wells were emptied and washed 3x with distilled water before 1 mL of 0.1% crystal violet solution (Sigma Aldrich) was added to each well and allowed to incubate for 20 minutes. The wells were emptied, washed again 3x with distilled water, and dried for 1 hour at room temperature. 1 mL/well of 95% ethanol was used to solubilize the remaining crystal violet and the absorbance of this solution was measured with a plate reader at 595 nm.

### Mammalian cytotoxicity

Adipose-derived stem cells (ADSCs) and fibroblasts (FBs) from human and mouse lineages were chosen to determine cell cytotoxicity in a variety of cellular profiles applicable to dermal wounds (Cell Application, San Diego, CA). ADSCs and FBs were cultured in cell-specific growth media to 95% confluency before seeding into a 12-well plate at 5 x 10^5^ cells/well. Wells were imaged to ensure equivalent densities of cells in each well. Media was aspirated from each well and 100 μL of 100X MIC gentamicin (500 mg/L) and clindamycin (100 mg/L) cHG+abx was pipetted directly on top of the cells. 200 μL of media was added to each well and the samples were incubated at 37° C for 10 minutes to induce gelation. Post-gelation, 1.8 mL of media was added to each well and the samples were incubated for up to 72 hours. As control, hFB cells were treated with cHG-only for up to 72 hours in the same manner. Post treatment, cells were stained with calcien AM and ethidium homodimer III (Thermo Fisher Scientific) and visualized using a KEYENCE BZX700 florescence microscope to quantify mammalian cell death. Fiji software was used to quantify cell death / viability post-treatment.

### In vitro scratch assay

*In vitro* scratch assays were completed following modified protocol of the established assay [26]. Human fibroblasts were seeded at 5 x 10^5^ cells/well in a 12-well dish using antibiotic-free media. After reaching 90% confluency, a 200 μL pipet tip with light suction was used to create a linear scratch through the cell monolayer. Cells were washed to remove excess cellular debris and media was replaced; scratch width measurements were taken via brightfield microscopy. The following groups were used: no infection / no treatment (control), no infection + cHG, infection + no treatment, infection + cHG, and infection + cHG+abx. For infection conditions, 50 μL of 10^6^ liquid MRSA culture was pipetted across the entirety of the scratch and allowed to attach for 2 hours at 37° C. The media was aspirated, and the scratch was treated topically with 100 μL of cHG or gentamicin cHG+abx (100X MIC, 500 mg/L). 200 μL of media was added to each well and the samples were incubated at 37° C for 10 minutes to induce gelation. Following gelation, 1.8 mL of media was then added to each well and the samples were placed again in incubator conditions (37° C). Infection conditions were compared against the no infection / no treatment and no infection + cHG groups. At 6, 12, 24, and 48 hours after treating the cells with cHG or cHG+abx, mammalian cells were stained with LIVE/DEAD stain, bacteria were stained with SYTO 61 stain, and the samples were imaged. Cell density within the original scratch area was quantified to measure mammalian cell migration. C. *perfringens* was not used in an *in vitro* scratch assay due to its anaerobic culture conditions.

### Statistical analysis

All statistical analysis was completed using GraphPad PRISM software (GraphPad Software, San Diego, CA, USA) and data were presented using mean ± standard deviation. Mean values were calculated from at least three independent experiments. Normalcy of longitudinal data collected at time points were verified with a Shapiro-Wilk test and histogram plotting; statistical significance was determined using a one-way analysis of variance (ANOVA) followed by Tukey’s post-hoc test. Data from different bacterial strains were not compared against each other. For the scratch assay, a two-way ANOVA followed by Tukey’s post-hoc test was used to test statistical significance. Statistical significance was established at p < 0.05.

## Results

### Scanning electron microscopy

Previous work has been done to characterize cHG prepared from the protocol used in this study [22]; SEM images were taken to ensure uniformity to the existing protocol. Collagen fibril formation was present with no under digested or over digested tendon visible in all samples imaged. Furthermore, collagen fibrils had no clear orientation, as expected, and collagen fibril cross-linkage was verified by the high degree of fibril interconnectivity.

### LCMS elution assay

**Fig 1:**
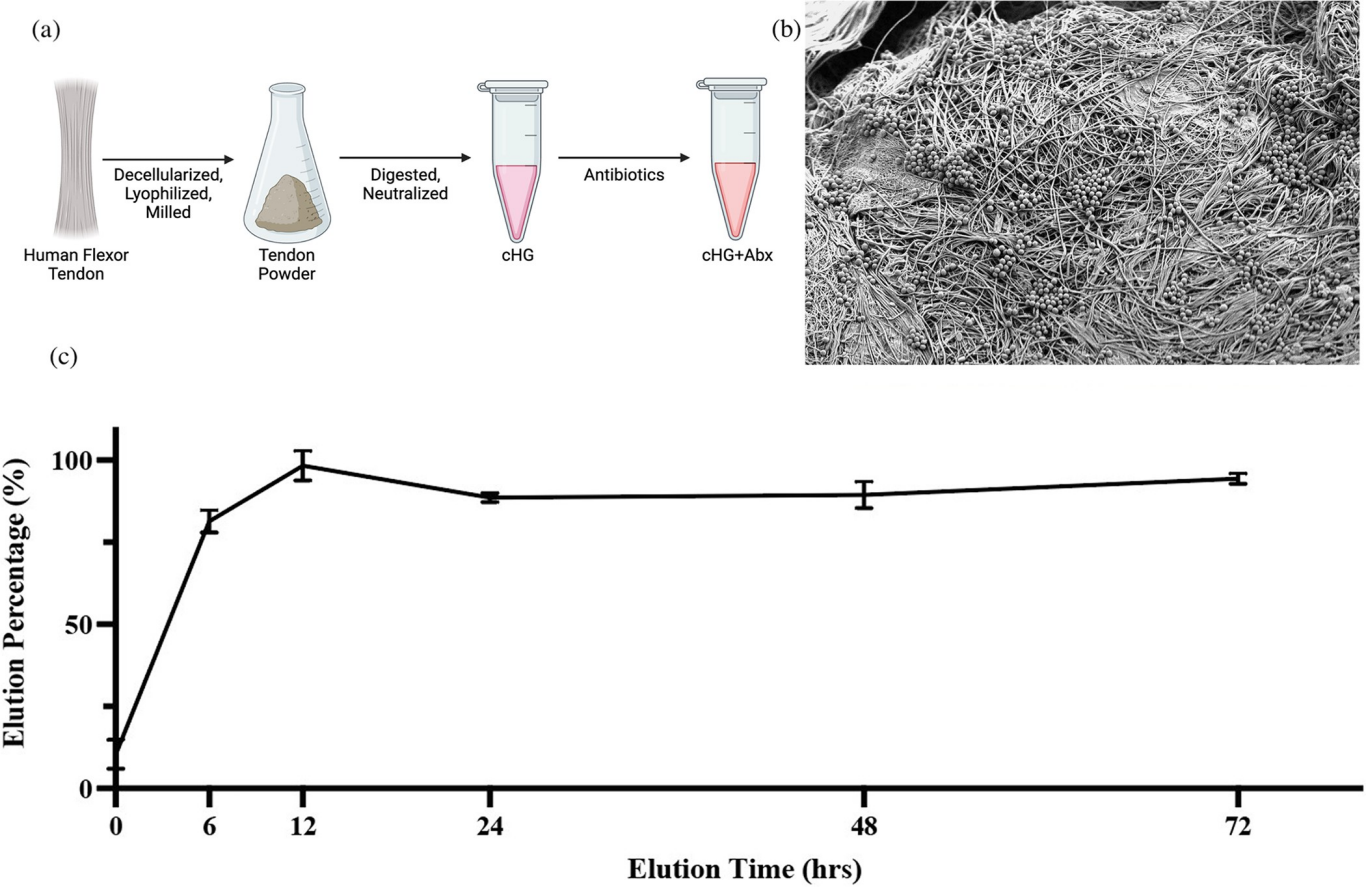
Overview of human-derived collagen hydrogel preparation and properties. (a) Complete process of cHG+abx fabrication. (b) SEM image of the surface of crosslinked hydrogel. (c) Pharmacodynamics of clindamycin elution from cHG over a 72-hour period measured by LCMS. Panel A created with BioRender.com.

cHG+abx eluate underwent liquid chromatography-mass spectrometry to determine the pharmacodynamic elution profile of the cHG+abx and potential antibiotic degradation occurring within the hydrogel, as antibiotic degradation is known to occur with changes to solution pH, temperature, or UV light exposure (Clindamycin, Fig 1C) [27, 28]. No degradation of clindamycin or gentamicin was observed at any time point up to 72 hours. Approximately 81% ± 3.3% of the antibiotic was observed to be released within the first 6 hours, followed by prolonged release for the following 6–18 hours.

### Modified Kirby-Bauer assay

**Fig 2:**
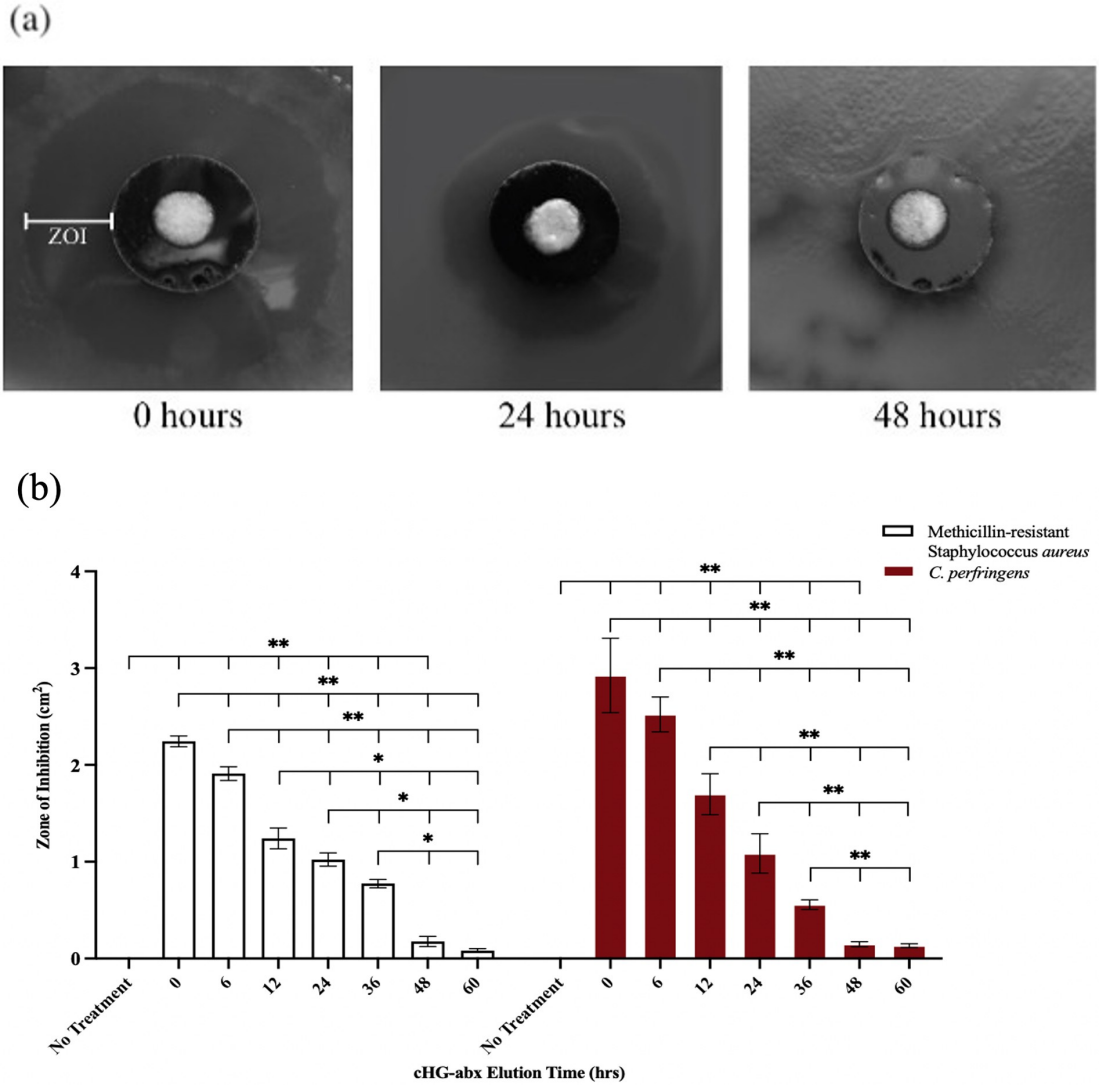
Modified Kirby-Bauer assay. (a) Representative images of the zone of inhibition (ZOI) after indicated hours of pre-elution. (b) Quantification of bacterial ZOI post-treatment with cHG+abx pre-eluted for up to 60 hours, compared to a no-treatment control. One-way ANOVA followed by Tukey’s test was used to establish significance. * indicates p-value < 0.05, ** indicates p-value < 0.01.

A modified Kirby-Bauer assay was completed to assess the ability of cHG+abx to elute inhibiting concentrations of antibiotic over time. Zones of inhibition (cm^2^) for both gentamicin cHG+abx treated MRSA and clindamycin cHG+abx treated *C*. *perfringens* are plotted in Fig 2 compared to a no-treatment group. Over the course of 48 hours of pre-elution, significant bacterial inhibition was observed for both bacterial strains at all time points. No significant inhibition was observed for 60 or 72 hours of cHG+abx pre-elution. For conditions treated with cHG+abx of 0, 6, 12, 24, 36, and 48 hours of pre-elution, a stepwise decrease in inhibition zone was noted at each period of pre-elution. No inhibition was observed in the cHG-only group.

### Biofilm inhibition visualization

**Fig 3:**
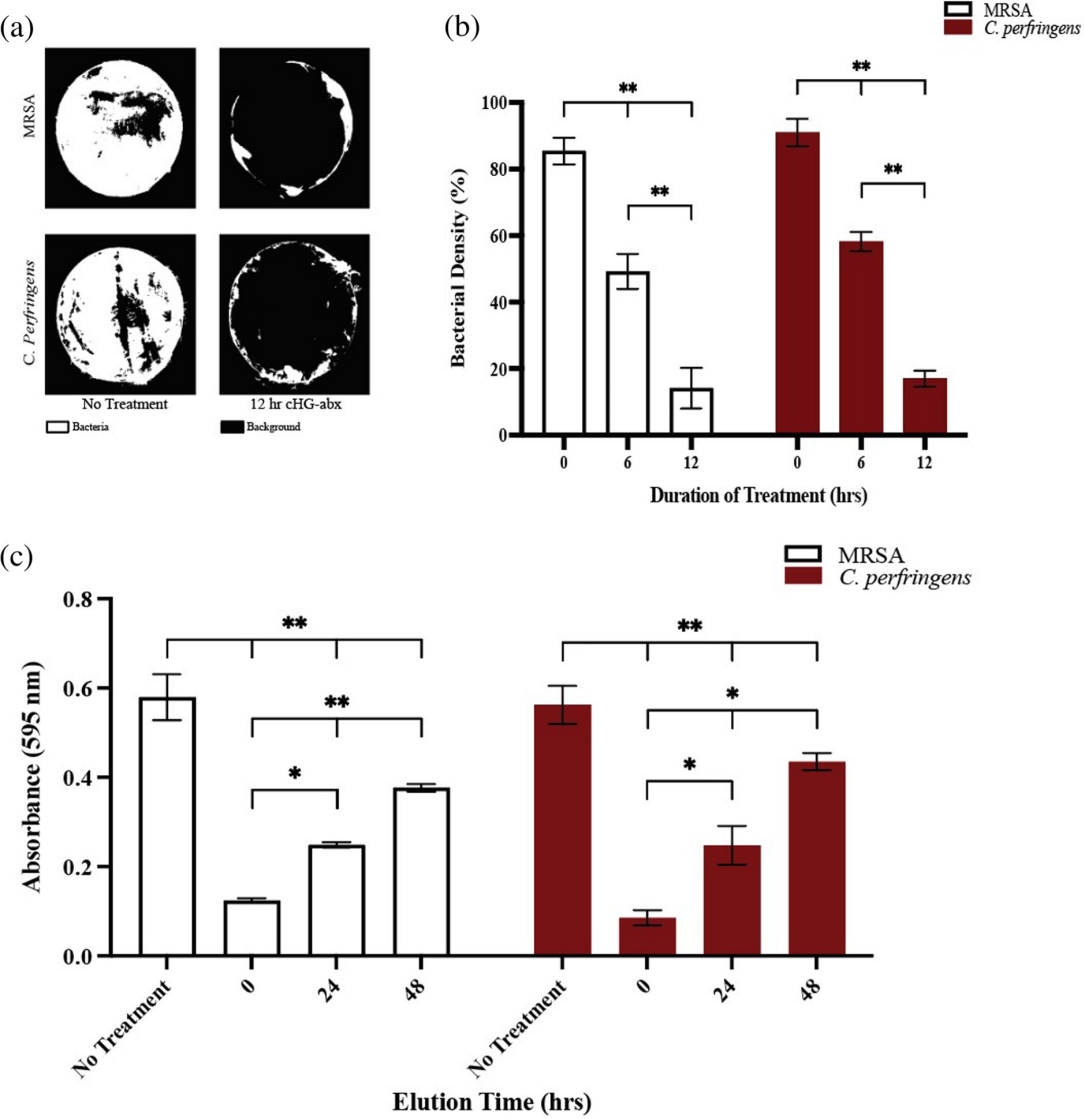
cHG+abx interference of bacterial biofilms. (a) Visualization of bacterial biofilms after 12 hours of topical treatment, compared to a no-treatment control. (b) Quantification of bacterial density stained with SYTO-61 in developed biofilms over 12 hours of topical treatment. (c) Absorbance (595 nm) of crystal violet solution from biofilms treated with cHG+abx pre-eluted for various timepoints, compared to control. One-way ANOVA followed by Tukey’s test was used to determine significance for both (b) and (c). * indicates p-value <0.05, ** indicates p-value < 0.01.

The efficacy and required time of cHG+abx treatment to significantly reduce the burden of biofilm-embedded bacteria was assessed with a standard biofilm inhibition assay. A significant stepwise decrease in bacterial activity, quantified by a decrease in fluorescence, was observed through fluorescent microscopy of treated MRSA and C. *perfringens* biofilms over the 12 hours of treatment (Fig 3A). MRSA and *C*. *perfringens* biofilms, observed at 6 hour into treatment, exhibited an average decrease in bacterial density of 42.7% and 37.7%, respectively. After 12 hours of treatment, the bacterial density decreases escalated to an average of 80.8% and 83.3%, respectively. Furthermore, at 12 hours of treatment, it was noted that all bacteria in direct contact with the cHG+abx had been eradicated, while only bacteria on the periphery of the membrane, not in contact with the hydrogel, survived. (Fig 3B). No inhibition was observed in the cHG-only control group.

### Crystal violet assay

A standardized crystal violet assay was conducted in conjunction with the biofilm inhibition assay to evaluate the timeline of antimicrobial concentrations of antibiotic elution from cHG+abx, with changes in crystal violet absorbance having a direct relationship with bacterial presence and viability post treatment. Biofilms treated with cHG+abx and quantified with crystal violet demonstrated significantly lower crystal violet solution absorbance for up to 48 hours of cHG+abx pre-elution for both MRSA and *C*. *perfringens*, with less absorbance correlating with decreased bacterial presence (Fig 3C). Furthermore, it was observed that there was a stepwise decrease in crystal violet solution absorbance (bacterial viability) correlating with hydrogel elution time. Specifically, in MRSA, the crystal violet solution absorbance was found to decrease by an average of 77.8%, 57.6%, and 36.3% when treated with cHG+abx with 0, 24, and 48 hours of pre-elution. Similarly, in *C*. *perfringens*, the absorbance decreased by 85%, 57.4%, and 21.4% when treated with cHG+abx with the same pre-elution durations. Both the no-treatment group and cHG-only control group demonstrated no significant reduction in crystal violet solution absorbance. No significant difference in biofilm disruption was observed between the no-treatment control, cHG-only, and cHG+abx membranes pre-eluted for 60 and 72 hours.

### Mammalian cell viability assay

**Fig 4:**
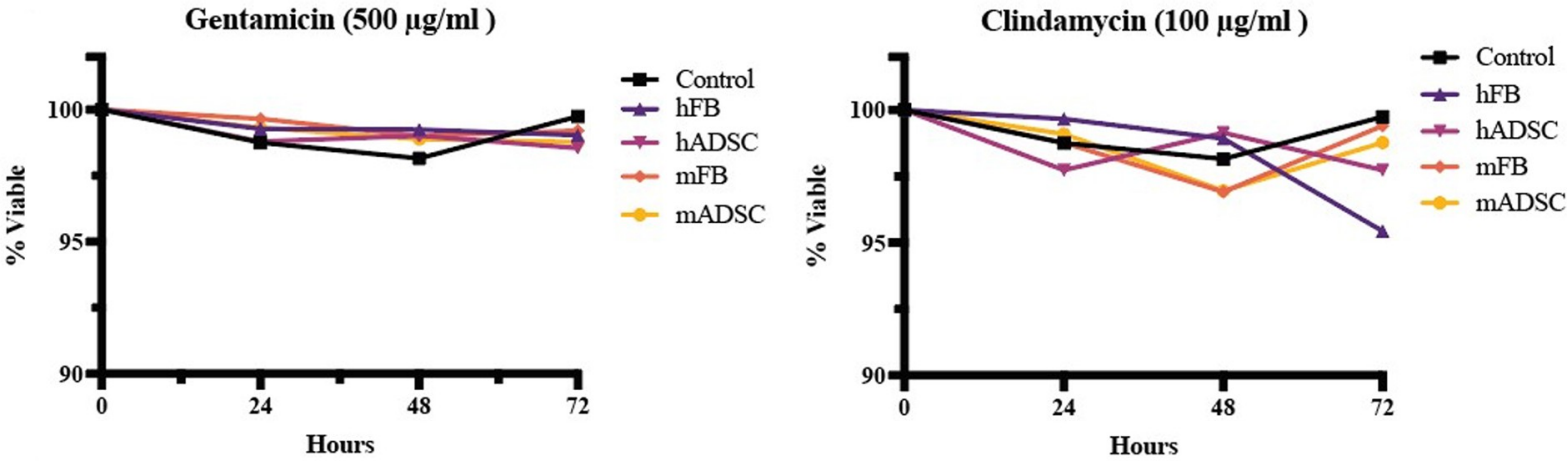
Mammalian LIVE/DEAD assay. LIVE/DEAD quantification of human and mouse cell lineages after up to 72 hours of topical treatment of cHG+abx, compared to a no-treatment control of hFB.

Since the antibiotic concentrations of cHG+abx used in this study were approximately 100X MIC higher than clinically administered orally or parenterally, mammalian cell viability was quantified post topical treatment with cHG+abx to determine the cytotoxicity of the treatment to mammalian cells. Following treatment with gentamicin cHG+abx, clindamycin cHG+abx, and cHG-only, quantifications of the ratio of viable mammalian cells to dead mammalian cells demonstrated that none of the treatment options resulted in significant cell death compared to a no-treatment control (Fig 4) at any time point up to 72 hours of treatment exposure. Furthermore, no morphological changes were observed at any timepoint in any cell linages.

### Modified scratch assay

**Fig 5:**
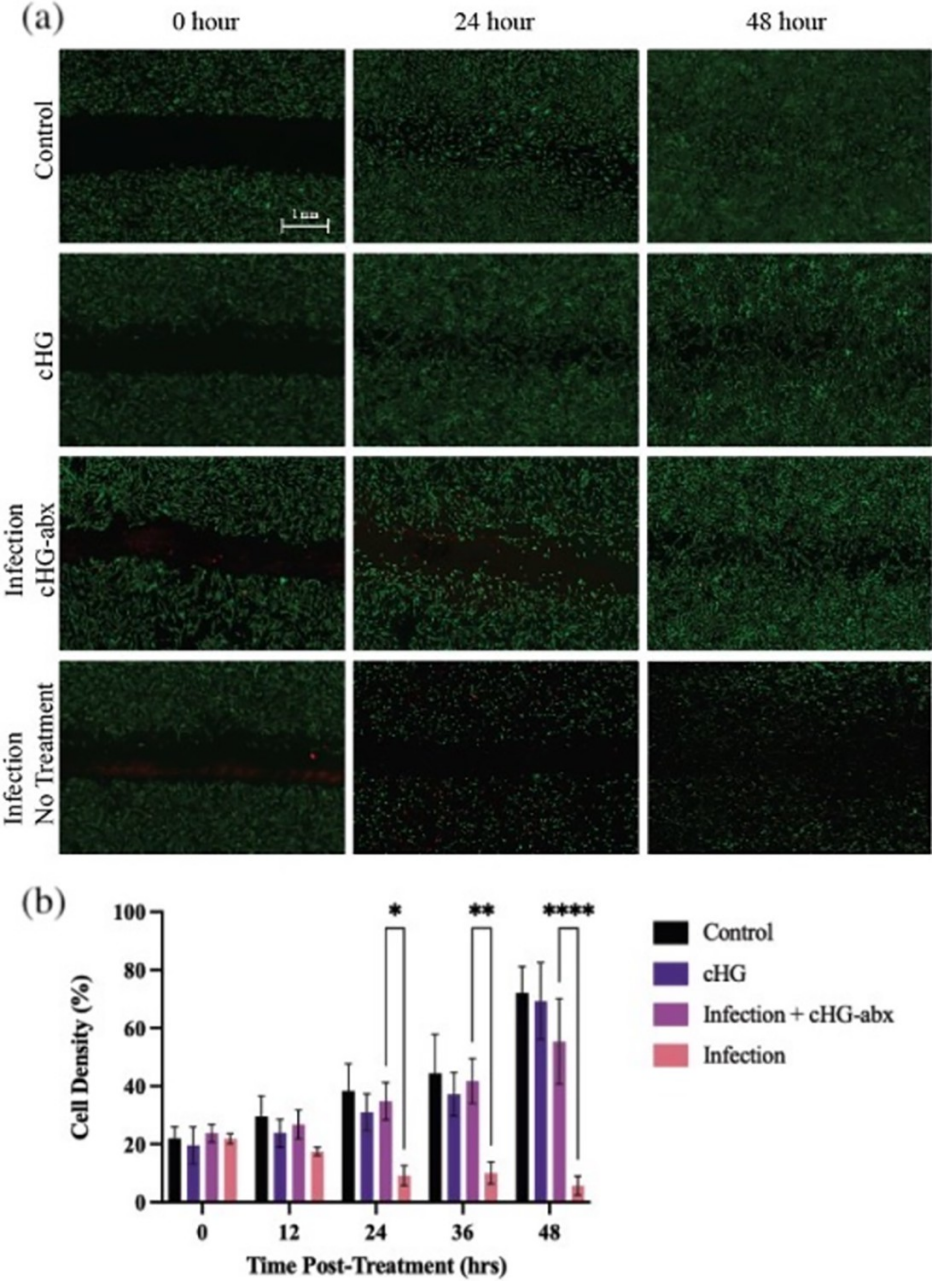
cHG+abx efficacy in an ex vivo fibroblast (FB) scratch model. (a) MRSA-colonized FB scratch assay imaged over 48 hours. Non-infected FBs treated with cHG alone (2^nd^ row) show similar growth compared to control (1^st^ row, untreated, non-infected FBs). FBs infected with MRSA without treatment (4^th^ row) showed significant FB death compared to infected FBs treated with cHG + abx (3^rd^ row). (b) Quantification of cell density within scratches of the modified scratch assay over 48 hours of migration. Two-way ANOVA with Tukey’s post-hoc test was used to establish statistical significance between experimental groups. * indicates p-value < 0.05, ** indicates p-value < 0.01, **** indicates p-values < 0.0001.

Bacteria-enhanced scratch assays were designed to mimic the natural use-case of cHG+abx to treat wounds using a rudimentary *in-vitro* model. The assay was used to determine if cHG+abx could both eliminate bacterial growth and sustain mammalian cell mobility. In the infection-only control and infection + cHG groups, mammalian cells co-cultured with bacteria showed widespread mammalian cell death, followed by eventual mammalian cell extinction. The no infection / no treatment control and no infection + cHG-only treated scratches demonstrated similar cellular migration speeds, reaching full closure by 36–48 hours, seen in Fig 5. Mammalian cells co-cultured with bacterial colonies topically treated with cHG+abx demonstrated bacterial growth inhibition by 24 hours, followed by full scratch closure at 48 hours. The cell density within the scratch was found to be significantly increased in the infection + cHG+abx group compared to the infection only group starting at 24 hours into treatment, continuing until the end of treatment. However, significant mammalian cell death was noted in the area surrounding the scratch in the infection only group. Cell density within the scratch was not significantly different between all no infection groups and the infection group treated with cHG+abx up to 48 hours.

## Discussion

Bacterial colonization and infection pose a significant challenge in the context of both chronic and unattended acute wounds, delaying wound healing and negatively impacting the patient. Addressing colonization at its source is crucial to minimize further bacterial growth but remains a formidable challenge for current therapeutic approaches [29]. In this study, we demonstrated that human-derived collagen hydrogel embedded with antibiotics effectively eluted inhibiting concentrations of antibiotic for methicillin-resistant *S*. *aureus* and *C*. *perfringens* biofilms for up to 48 hours with no deleterious effects on mammalian cells at antibiotic concentrations up to 100X the minimum inhibitory concentration. This study, in conjunction with our prior research [16, 30, 31], confirms the efficacy of human-derived collagen hydrogel as an antimicrobial dressing for targeting superficial wound bacterial biofilms.

The antibiotic release profile of the human-derived collagen hydrogel is well suited to treat superficial bacterial biofilms. The results of the Kirby-Bauer assay and crystal violet assay demonstrated the hydrogel successfully eluted bactericidal concentrations of antibiotic for both MRSA and *C*. *perfringens* grown in culture and in biofilm formation for up to 48 hours (Figs 2 and 3). Furthermore, fluorescent images taken of the biofilms during topical treatment demonstrated significant bacterial death at 6 hours into treatment and full bacterial eradication at 12 hours in areas of cHG+abx–bacteria contact (Fig 3A and 3B). Specifically examining the elution kinetics of antibiotic release from the hydrogel, LCMS testing demonstrated the hydrogel yields an initial burst release of approximately 81% of the embedded antibiotic within the first 6 hours, with antibiotics remaining in their clinically active form for an additional 60 hours. Furthermore, the initial burst release of high-dose antibiotics, as depicted in Fig 4, did not adversely affect mammalian cells. This rapid release profile may offer advantages in targeting dormant bacterial cells within biofilms. These biofilms are notorious for entrapping highly antimicrobial-resistant bacterial cells within their extracellular polymeric substances [12], hindering drug penetration. Moreover, surviving bacteria within biofilms can rebound with increased resistance after exposure to sub-lethal doses of antimicrobials [11, 32]. We anticipate that these findings will translate into effective eradication of naturally forming biofilms. The hydrogel’s ability to deliver a burst release of bactericidal antibiotics, alongside its sustained release kinetics, presents a promising strategy for combating both biofilm-embedded bacteria and preventing bacterial regrowth. Nevertheless, further validation through in vivo studies and clinical trials will be crucial to confirm its efficacy in real-world settings. To address the concern of repeated hydrogel application after 48 hours of use, our group is continuing to investigate methods of extending the total time of antibiotic release.

Our group also demonstrated that human-derived collagen hydrogel is a versatile carrier of various antibiotics and effective for the treatment of multiple biofilm phenotypes. We observed both MRSA and *C*. *perfringens* were successfully eradicated with hydrogel treatment containing gentamicin and clindamycin concentrations as low as 10X MIC (50 mg/L and 10 mg/L, respectively); the collagen hydrogel environment was suitable for gentamicin and clindamycin release and induced no antibiotic degradation. Moreover, prior work from our lab demonstrated human collagen hydrogel as a viable carrier for both ciprofloxacin and ceftazidime to successfully disrupt *Pseudomonas aeruginosa* biofilms at analogous antibiotic concentrations when applied topically [21]. This has important clinical implications as colonized cutaneous wounds typically have complex bacterial compositions that require personalized therapy [33]. The capacity for human-derived collagen hydrogel to carry a wide variety of antibiotics and treat multiple clinically significant bacterial phenotypes is strong support for their use as a practical, customizable option for dermal wounds. Further studies investigating treatment of multi-species biofilms are underway [33, 34].

A specific benefit of using collagen hydrogels over other frequently used hydrogel polymers is collagen’s intrinsic role in the wound healing process [35]. Use of collagen hydrogels has been reported to increase cell adhesion and migration speed in *in vitro* models [36, 37]. Using a modified scratch assay, we demonstrated the introduction of bacteria into the scratch led to inhibited mammalian cell migration, change in mammalian cell morphology, and widespread mammalian cell death. However, when bacteria-containing scratches were treated with cHG+abx, mammalian cells demonstrated initial slowed migration during bacterial eradication followed by recovery of migration to rates comparable to non-infected controls. Furthermore, mammalian cells treated with cHG demonstrated a degree of three dimensionality, indicating cell attachment to the hydrogel. Cell attachment to the hydrogel *in vivo* may enhance cell migration into the wound space to speed wound closure. *In vivo* models have established the wound-regenerative properties and cosmetic utility of collagen hydrogels; collagen hydrogels have been shown to enhance granulation tissue deposition, increase the rate of angiogenesis, re-epithelialization, and wound remodeling, and reduce wound contraction and scarring [38–40]. By leveraging collagen’s wound-regenerative properties and its capacity as an antimicrobial carrier, topical treatment with embedded collagen hydrogels offers a comprehensive method for addressing the bacterial burden and impaired healing capacity observed in colonized wounds, addressing key limitations of current wound management strategies.

Human-derived collagen polymers serve a dual purpose, acting as a reliable and adjustable polymer for the delivery of various pharmaceuticals, while also serving as a biocompatible scaffold with therapeutic benefits [19, 36, 37]. In comparison to synthetic polymers, organic polymers offer several advantages, including biocompatibility, biodegradability, and bioactivity. Nevertheless, they lack the level of customization achievable with synthetic polymers [41]. Synthetic polymers can be specifically designed to exhibit tailored release profiles and mechanical properties [42, 43], which would be highly advantageous for extending the duration of antibiotic release, as discussed in within this work [44]. However, prolonged use of synthetic polymers carries the risk of toxicity and does not offer the same therapeutic benefits as organic polymers [43, 45]. Other organic polymers, such as fibrin, dextran, and alginate have comparable biocompatibility and biodegradability to collagen polymers. However, non-collagen polymers possess inherent limitations when used topically and fail to provide the same therapeutic advantages as collagen hydrogels in cutaneous wound healing [45, 46]. To attain similar customizability to synthetics in organic hydrogels, various engineered features, such as embedded pharmaceutical carriers, pharmaceutical-scaffold covalent bonding, and composite hydrogels, have been designed [42, 47, 48]. The stem cell homing [49], wound healing [50], and antibiotic delivery characteristics of cHG make it an ideal hydrogel for further enhancement to tackle biofilms and other infected wounds [21, 51].

This study is limited by using single-species biofilms. Colonized cutaneous wounds are rarely colonized by a single species of bacteria; in fact, most colonized wounds contain multiple species of bacteria, as well as fungi, algae, and protozoa [52, 53]. To optimize the physiological accuracy of *in vitro* biofilm models, future studies are planned address treatment effectiveness against multi-species biofilms. Another limitation of this study is the limited timespan of effective pharmaceutical activity. Though the benefits of the initial antibiotic burst release, followed by continued effective antibiotic activity, are discussed above, extending the total timeframe of pharmaceutical release would lengthen the duration of bacterial inhibition and reduce the required frequency of dressing changes for patients. Work is underway to address these limitations.

## Conclusion

Overall, this study demonstrates the capacity of human collagen hydrogel to provide high concentration, sustained antibiotic release resulting in bacterial inhibition and biofilm disruption while maintaining mammalian cell viability. The work completed in this manuscript translates clinically. These findings broaden the applications of human-derived collagen hydrogel and support its use as a topical treatment option for colonized wounds. Furthermore, this treatment, whether used in combination with standard approaches or as a standalone therapy, directly targets bacteria and biofilms. Widespread cHG+abx usage has the potential to mitigate adverse side effects linked to conventional treatments while providing improving wound healing outcomes.
